# Parallel Mechanisms for Re-Epithelialization Following Skin Cell Suspension Autograft Application: Scientific Insights into Acute Wound Healing

**DOI:** 10.1093/jbcr/iraf219

**Published:** 2025-11-28

**Authors:** Katie A Bush, Elisa N Stephens, Ghaidaa Kashgari, Aleisha K Chamberlain, Steven A Kahn

**Affiliations:** AVITA Medical, Valencia, CA 91355, United States; AVITA Medical, Valencia, CA 91355, United States; AVITA Medical, Valencia, CA 91355, United States; AVITA Medical, Valencia, CA 91355, United States; Department of Surgery, Medical University of South Carolina, Charleston, SC 29425, United States

**Keywords:** skin regeneration, re-epithelialization, autologous skin cell suspension, wound healing, autografting

## Abstract

Timely closure of acute, full-thickness wounds is critical in minimizing complications such as infection, fluid loss, and impaired healing, all of which can adversely affect long-term patient outcomes. Although meshed autografting is the current standard of care, its effectiveness is limited by the need for donor skin and the re-epithelialization of expanded interstices. Prior research has shown that combining meshed autografts with skin cell suspension autograft (SCSA) enhances epidermal regeneration. In this study, we further investigate the mechanisms by which SCSA promotes re-epithelialization when applied with a widely expanded (3:1) meshed autograft in a full-thickness porcine wound model. Histological analyses demonstrate complete closure of graft interstices as early as 3 days post-surgery. A dual mechanism of re-epithelialization was observed, with keratinocytes migrating both from the edge of the interstices and from within the center of the interstices to form a continuous epithelial layer. The presence of a high number of proliferating cells in the wound bed further supports the regenerative activity of SCSA. These findings offer valuable mechanistic insight into the role of SCSA in accelerating wound closure and provide additional evidence for its use in improving outcomes for patients with acute full-thickness wounds.

## INTRODUCTION

Early closure of full-thickness wounds is critical to ensure positive patient outcomes. During the healing phase, the compromised barrier function leads to excessive fluid loss and heightened vulnerability to pathogens, which can contribute to patient complications and prolonged length of stay (LOS).^1^ Increased time to closure has been shown to impact scar quality, with longer healing times significantly increasing risk of hypertrophic scarring.^2^^,^^3^ Hypertrophic scarring can result in poor esthetic outcomes and impaired functional outcomes, especially in cases in which scarring extends across a joint.^4^ For these reasons, minimizing time to wound closure remains an undisputed goal of acute therapeutic wound care.

The current standard of care for full-thickness acute wounds, such as those caused by burns and non-thermal trauma, consists of surgical excision followed by grafting with autologous donor skin. Although effective, autologous split-thickness skin grafts (STSGs) are associated with significant donor site morbidities, including pain, infection, and scarring, and are limited by the availability of uninjured skin.^5^^,^^6^ Though meshing donor skin allows STSGs to expand and provide more coverage, thus, reducing donor skin requirements, higher meshing ratios necessitate greater migration of keratinocytes to re-epithelialize, which can prolong healing time.^7^

A device-prepared, point-of-care skin cell suspension autograft (SCSA) has been established as an effective treatment for burns and non-thermal, full-thickness wounds to address limitations of standard autografting procedures. Primarily comprised of keratinocytes, fibroblasts, and melanocytes isolated from a small split-thickness piece of uninjured skin, SCSA is applied to properly prepared wounds to promote epidermal regeneration.^8^ When used in combination with meshed STSGs (mSTSGs), SCSA has been shown to significantly reduce donor skin requirements compared to mSTSG alone without compromising healing outcomes or safety, when compared to mSTSGs meshed by 1 factor less.^9^^,^^10^

In a recent study evaluating its biological attributes, SCSA was found to contain a notable population of activated keratinocytes, which are known to play a central role in re-epithelialization.^11^ Following injury, healthy keratinocytes at the wound edge are activated by interleukin-1, leading to changes in the regulation of genes involved in cytoskeleton composition, receptor expression, proliferation, and migration to promote wound closure.^12^^,^^13^ Importantly, keratinocyte activation is maintained through the secretion of autocrine and paracrine signaling factors from these cells until a keratinocyte monolayer covering the entire wound bed is achieved. In addition to containing these activated keratinocytes, SCSA was also shown to be primarily comprised of non-aggregated single cells, which are non-contact inhibited and able to experience a “free-edge” signal that can increase migration velocity from the wound edge.^14^ These inherent biological attributes make the cells in SCSA uniquely primed for wound healing. Application of SCSA to human dermal equivalents indicated functional cells able to produce a mature, stratified epidermal layer within 3 weeks with actively proliferating cells present at the dermal-epidermal junction, as is expected for healthy re-epithelialization trajectories.^11^

In addition to cell-based evidence, other studies have elucidated the wound-healing properties of SCSA using animal models. In an early head-to-head study conducted by Navarro et al., porcine wounds treated with 3:1 meshed autograft and SCSA healed faster and showed a better quality of re-epithelialization than wounds without SCSA.^15^ In particular, histologic analysis revealed treatment with SCSA contributes to the formation of a more robust epithelial layer with greater thickness and vascularization. Similarly, Collins et al. recently showed that wounds treated with 4:1 meshed autograft and SCSA exhibited increased rates of wound healing and increased epidermal thickness at early timepoints compared to wounds that did not receive SCSA.^16^ Using digital planimetry, it was found that wounds treated with SCSA re-epithelialized earlier for both burn and excisional conditions. Taken together, these findings support the regenerative capacity of the keratinocytes within SCSA and further suggest that accelerated wound healing occurs at the cellular level.

Questions have been raised regarding the rate of re-epithelialization with SCSA. In clinical use, it is noted that a translucent sheen across graft interstices is present in the early healing phase, indicative of initial re-epithelialization, but understanding what this represents remains to be confirmed at the histological level. Given the evidence supporting the role of SCSA in enhancing epidermal regeneration, understanding the underlying mechanisms behind these effects would be highly advantageous.

The purpose of this study was to evaluate *in vivo* epidermal regeneration of wounds treated with SCSA, specifically investigating its effects on full-thickness wounds treated with mSTSGs at the level of the interstice. Porcine wound healing models are generally considered to be the most appropriate animal model for assessing the clinical efficacy of therapeutic interventions as porcine skin has comparable epidermal thickness and wound healing properties compared to those of human skin.^17-20^ By evaluating healing outcomes of interstices at early timepoints using this model, this study provides insight into the mechanisms by which SCSA achieves accelerated healing, further supporting its use to achieve more timely healing of acute wounds.

## MATERIALS AND METHODS

### Animal model

Six female Yorkshire pigs (3 per timepoint) aged approximately 3 months (20-25 kg) were obtained for this study. Prior to study start, animals were acclimated for 7-10 days in a facility accredited by the American Association for the Accreditation of Laboratory Animal Care. This study received Institutional Animal Care and Use Committee approval.

### Surgical procedure

**Figure 1:**
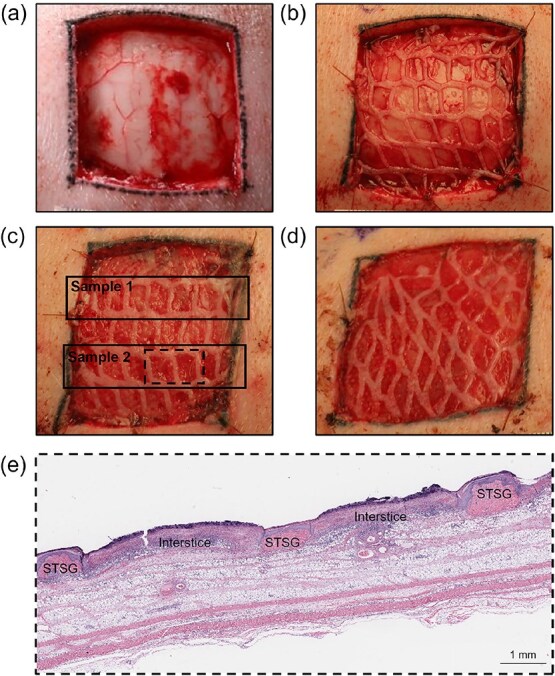
Study Timeline and Design. (a) Representative photographs of 4 cm × 4 cm wounds at (a) Day 0 (post-excision), (b) Day 0 (post-mSTSG/SCSA treatment), (c) post-operative Day 3, and (d) post-operative Day 6. (e) Representative histological image of wound bed section harvested on Day 3 and stained with H&E, showcasing multiple interstices within 1 sample.

All surgical procedures were carried out under proper sedation and pain relief. Prior to surgery, the hair on the dorsum and the flank of the pig was shaved, and the areas were scrubbed with germicidal soap and prepared for surgery. To minimize chemical interactions, no chemical hair removal products were used. Full-thickness wounds (4 cm × 4 cm) were created using a scalpel on the dorsum on each side of the midline parallel to the spine by surgical excision, removing the entire epidermis and dermis, along with the fat layer above the fascia (Figure 1a).

Autografting was performed immediately following full-thickness surgical excision (Figure 1b). The area of skin required for mSTSGs and SCSA was harvested using a dermatome (Zimmer, Warsaw, IN). Donor skin samples were collected from the flanks of the animals at a depth of 0.010-0.012” for mSTSGs and 0.006-0.008” for SCSA. Skin allocated to mSTSG was prepared at a meshing ratio of 3:1 using a standard skin graft mesher (Zimmer, Warsaw, IN). All meshed and non-meshed donor skin was kept moist in gauze and soaked in sterile saline until use. All wounds received a 3:1 mSTSG, secured in place using sutures.

### Skin cell suspension autograft preparation

Skin cell suspension autograft was prepared using the RECELL^®^ System (AVITA Medical, Valencia, CA). Briefly, SCSA donor skin samples were incubated in the proprietary enzyme solution for 30 minutes and subsequently washed with buffer. Digested skin samples were assessed for epidermal/dermal separation and scraped vigorously until the epidermis was in suspension and the dermis had disintegrated. The resulting suspension was filtered through a strainer before use.

### Skin cell suspension autograft application

Following grafting, SCSA was applied dropwise (400 μL total per wound) over the entire surface of all wounds at a seeding density of 1.24 × 10^5^ ± 0.22 × 10^5^ viable cells/cm^2^. A Telfa™ non-adherent clear wound dressing (Medtronic, Minneapolis, MN) was immediately applied as the primary dressing to minimize suspension run-off following SCSA application and to protect the wound bed. Wounds were dressed with Xeroform™ petroleum gauze dressing (Medtronic, Minneapolis, MN), Mextra Superabsorbent dressing (Mölnlycke Health Care, Peachtree Corners, GA), occlusive drape V.A.C.^®^ (M6275009, KCI-USA, Arlington, TX), and Elastikon tape (Johnson & Johnson, New Brunswick, NJ). Dressed wounds were fixed with an abdominal jacket. Dressing changes were performed under sedation at 72 hours (± 24 hours).

### Transepidermal water loss

TEWL measurements were collected using the Tewameter^®^ TM Nano probe (Courage + Khazaka, Köln, Germany) with 3 measurements taken for each wound, as well as for control uninjured tissue at 3- or 6-days post-surgery.

### Biopsy acquisition

Biopsy specimens were collected at terminal time points (3- or 6-days post-surgery), during which the entirety of the wound bed was harvested with at least 0.5 cm of additional, uninjured skin from the edge of each wound (Figure 1c and d). Each wound was subsequently cut into 2 pieces, each containing an independent row of interstices and healthy tissue margin (approximately 6 interstices per row). The collected samples were placed in 10% neutral-buffered formalin and sent for paraffin embedding and staining.

### Histopathology

Formalin-fixed biopsy samples were embedded in paraffin and sectioned at 4 μm. Two-step sections were taken from each block to assess re-epithelialization, stratification, and proliferation at multiple wound levels (approximately 6 interstices per section, 12 per sample, and 24 per wound). Serial slides were stained with Surgipath^®^ Harris hematoxylin and eosin (Leica Biosystems, Deer Park, IL), pancytokeratin, or anti-Ki-67 antibody secondary counterstains. Whole-slide scanning (40x) was performed on a Leica Aperio AT2 digital pathology scanner.

### Quantitative assessments

To assess wound healing, percent re-epithelialization, epidermal thickness, and cell proliferation were measured at each time point by a blinded, certified third-party pathologist using histological evaluation. For each wound, all captured interstices were assessed (Figure 1e). Percent re-epithelialization was calculated as the total length of the epithelium normalized to the interstice width. Epidermal thickness measurements were collected from 6 different regions of each interstice (2 from the left, 2 from center, and 2 from right) and averaged. Cell proliferation was assessed by the number of epidermal Ki67+ cells counted per 40x Grid and reported per mm^2^.

Average values for all assessments were calculated from individual interstice measurements per wound and were used to calculate overall means (*n* = 6 per timepoint).

### Statistical analysis

Two-tailed correlation analyses were performed for evaluating the relationship between interstice width and percent re-epithelialization 3- or 6-days post-application of SCSA. *P* < .05 was used as a threshold for statistical significance. Statistical analyses were performed using GraphPad Prism (GraphPad Software).

## RESULTS

**Figure 2:**
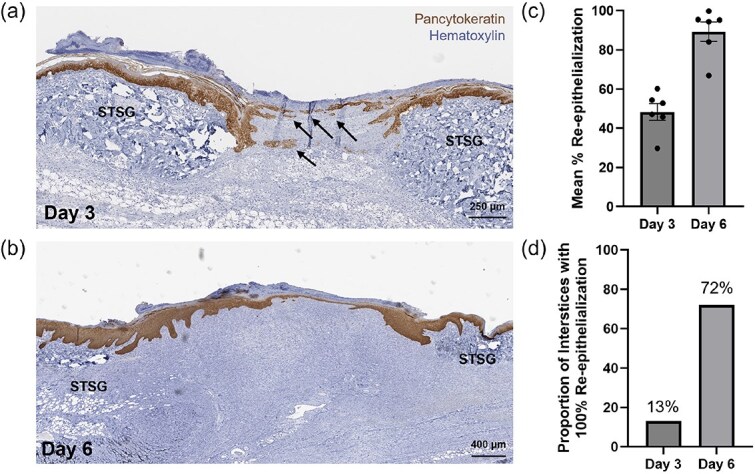
Re-Epithelialization with Skin Cell Suspension Autograft at the Cellular Level. Representative images of tissue biopsies taken at (a) Day 3 and (b) Day 6 stained with hematoxylin and pancytokeratin to assess keratinocyte migration over the study timeline. Arrows indicate formation of keratinocyte islands at Day 3. Histological evaluation of wound beds was performed to calculate (c) the average percent re-epithelialization ± SEM at Day 3 and Day 6 as determined by wound means (*n* = 6) and (d) the proportion of interstices that achieved complete (100%) re-epithelialization at Day 3 and Day 6 post-skin cell suspension autograft application.

Pancytokeratin staining of tissue biopsies from the wound bed revealed the formation of distinct keratinocyte islands within the skin graft interstices at Day 3 and often a complete keratinocyte layer spanning the interstices by Day 6 (Figure 2a and b). Quantification of re-epithelialization demonstrated that, on average, interstices were 48% re-epithelialized at Day 3 and 89% re-epithelialized at Day 6 following treatments with SCSA (Figure 2c). Notably, 13% of interstices measured at Day 3 and 72% of interstices measured at Day 6 achieved complete (100%) re-epithelialization (Figure 2d).

**Figure 3:**
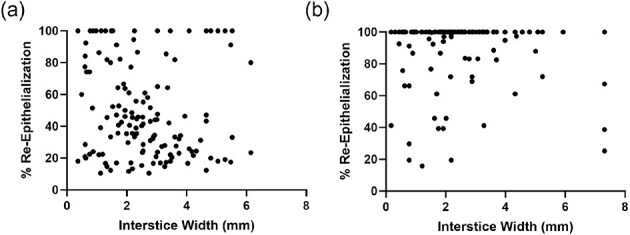
Impact of Interstice Width on Percent Re-Epithelialization with Skin Cell Suspension Autograft. Scatter plots showing distribution of technical replicates by width and percent re-epithelialization at (a) Day 3 and (b) Day 6 following applications of a skin cell suspension autograft. Correlation analysis reveals Pearson r = -0.103 at Day 3 (*P* = .235) and Pearson r = -0.231 at Day 6 (*P* = .006).

The overall mean interstice width ± SEM [average minimum, average maximum] for wounds evaluated on Day 3 and Day 6 were 2.60 ± 0.14 mm [1.96, 3.02 mm] and 2.45 ± 0.20 mm [1.86, 3.20 mm], respectively. Evaluation of the effect of interstice width on percent re-epithelialization using the total amount of technical replicates for each timepoint (*n* = 135 interstices for Day 3 and *n* = 141 interstices for Day 6) via scatter-plot analysis demonstrated no significant correlation between interstice width and percent re-epithelialization at Day 3 (Pearson r = -0.103, *P* = .235) and a weak inverse correlation at Day 6 (Pearson r = -0.231, *P* = .006) (Figure 3).

**Figure 4:**
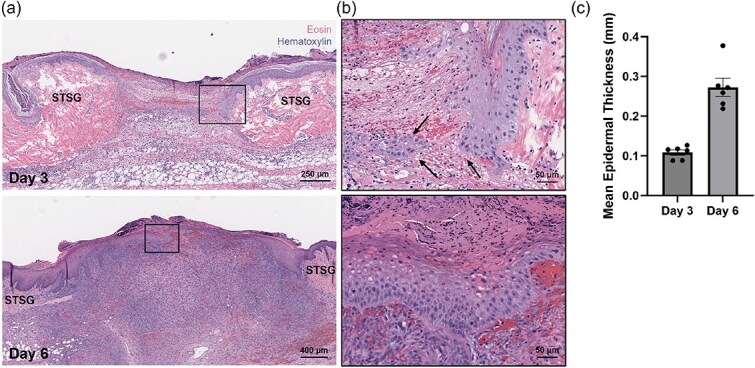
Epidermal Regeneration with Skin Cell Suspension Autograft at the Cellular Level. Representative images of histological staining at Day 3 and Day 6 using H&E at (a) the level of the interstice and (b) at the cellular level. (c) Resulting quantification of epidermal thickness measurements at both days displayed as overall means ± SEM. Arrows indicate cellular repopulation from both the interstice edge and within the center of the interstice.

Histological analysis stained with H&E showed a greater density of cells within the interstice at Day 6 compared to Day 3 (Figure 4a). Further, images taken at a greater magnification indicated epidermal cells repopulating the interstice both from the edge of the interstice and from the center of the interstice (Figure 4b). The mean epidermal thickness ± SEM of the observed epidermal layer was 0.11 ± 0.01 mm at Day 3 and 0.27 mm ± 0.02 mm at Day 6 (Figure 4b), representing a 2.5-fold increase consistent with qualitative assessments.

**Figure 5:**
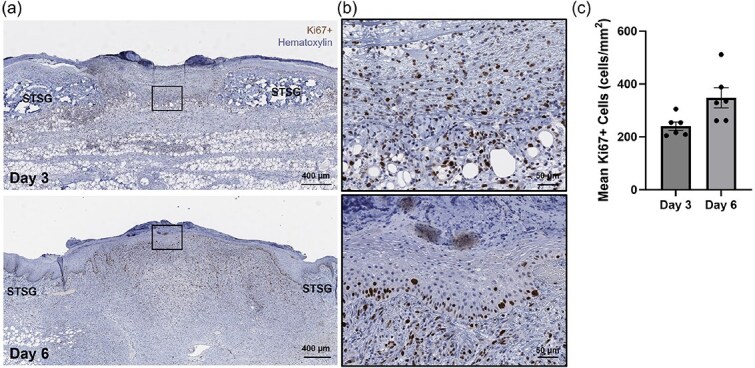
Localization of Proliferative Cells Post-Skin Cell Suspension Autograft Application. Representative images of histological staining using hematoxylin and anti-Ki67 antibody at (a) the level of the interstice and (b) at the cellular level. (c) Resulting density of Ki67+ cells measured at both days displayed as overall means ± SEM.

Immunohistological staining for Ki67, a marker for proliferating cells, indicated widespread cellular proliferation throughout the wound bed at Day 3, with these proliferative cells predominantly localized to the dermal-epidermal junction by Day 6 (Figure 5a and b). The mean density ± SEM of Ki67+ cells in the wound beds increased from 240 cells/mm^2^ ± 16 cells/mm^2^ at Day 3 to 348 cells/mm^2^ ± 38 cells/mm^2^ at Day 6, reflecting a 1.4-fold increase in proliferative activity (Figure 5c).

Prior to wound bed harvesting, mean transepidermal water loss (TEWL) measurements ± standard deviation were 90.6 ± 7.3 g/m^2^/h for day-3 wounds and 89.4 ± 4.7 g/m^2^/h for day-6 wounds, while the TEWL measurement of uninjured skin was 22.3 ± 4.0 g/m^2^/h.

## DISCUSSION

Timely re-epithelialization of wounds following injury is critical for restoring homeostasis and reducing risk of infection and adverse scar formation.^1-3^ Skin cell suspension autograft is an advanced treatment option that leverages the regenerative capacity of keratinocytes to promote wound closure, while minimizing donor skin requirements.^8-11^ In this study, epidermal regeneration following SCSA application over meshed skin grafts was evaluated at the level of individual interstices to understand how the cell suspension contributes to re-epithelialization of full-thickness wounds.

Histological analysis revealed that re-epithelialization occurs as early as 3 days post-surgery, with 13% of evaluated interstices achieving complete re-epithelialization. Epidermal thickness increased from Day 3 to Day 6, with multiple cell layers present, and localization of proliferative cells to the dermal-epidermal junction was observed by Day 6.

This study reaffirms previous reports of initial rates of healing and provides further insight into the mechanism by which the cells in SCSA achieve this outcome. Although a major limitation is the lack of a control arm, this study falls in line with previously reported values from animal studies evaluating treatment with mSTSG + SCSA and control groups lacking SCSA. In Collins et al., wounds treated with SCSA re-epithelialized significantly faster than untreated controls.^16^ By Day 5, re-epithelialization in burn wounds was 86% for SCSA-treated wounds versus 60% for control-treated wounds. In Navarro et al., re-epithelialization in excisional wounds at Day 5 was 82% versus 71% for SCSA-treated group compared to control, respectively.^15^ In the current study, re-epithelialization rates are comparable to the treatment groups from the previous reports, with an average of 89% at Day 6. Increased epidermal thickness was also found to be increased in both studies compared to controls, correlating with the epidermal thickness outcomes observed in this study.

Characterization of healing in the present study suggests a dual mechanism of re-epithelialization with cells at the margins of the interstices and cells from SCSA both contributing to epidermal regeneration, as evidenced by migration of the epidermal tongue from the healthy skin graft into the interstices and formation of keratinocyte islands in the center of the interstices at Day 3. Although not specifically tagged, the cells present in the interstices are hypothesized to be derived from SCSA, as the wounds in this study were excised to full thickness, resulting in exposed muscle fascia and removal of all viable dermal appendages.

When assessing re-epithelialization, we observed regions of non-continuous epithelial coverage, an outcome that, to our knowledge, has not been previously described. Scatter plot analyses further demonstrated substantial variability in re-epithelialization across interstice widths at Day 3. Importantly, no significant correlation was detected between interstice width and percent re-epithelialization at Day 3, and only a weak inverse correlation emerged by Day 6 (*P* = .006). These findings indicate that interstice size alone does not govern re-epithelialization dynamics. Instead, the data supports a dual mechanism of closure in which applied cells actively populate and bridge wider gaps, while resident keratinocytes migrate inward from the wound margins. If closure were driven exclusively by edge-derived keratinocytes, a strong, consistent correlation between interstice width and re-epithelialization would be expected.

The TEWL measurements presented in the current study are 3 times higher than that of native skin, which suggests a weaker barrier function relative to uninjured skin, but supports the formation of an immature epidermis at these early time points. Histological sections also confirm these data, as an immature stratum corneum is present. From a clinical perspective, this finding highlights the importance of protecting the wound bed with an appropriate dressing post-SCSA application to prevent shearing of the newly formed epidermal layer and to protect the wound from excessive fluid loss until maturity is obtained.

In addition to expanding the breadth of pre-clinical evidence related to the mechanism of healing when SCSA is incorporated into the treatment algorithm, this study supports clinical histological findings. In a study by Hultman et al., histological analysis of punch biopsies obtained from a full-thickness laceration treated with a 3:1 mSTSG and SCSA revealed the presence of a neo-epidermis 6-8 keratinocytes thick just 1-week post-surgery, with abundant rete ridges indicative of healthy, uninjured skin present by 6 weeks.^21^

While this study offers valuable mechanistic and clinical insights, several limitations should be noted. First, the study was uncontrolled, relying on existing preclinical data for indirect comparisons. It was further limited by a small number of unique animal subjects. Although the porcine model is a well-established proxy for human wound healing, important differences remain, including reduced inflammatory responses and variations in tissue repair processes compared to human thermal burns. Furthermore, this study was limited to young female animals and did not evaluate the impact of sex on healing. Despite this, previous studies evaluating SCSA using exclusively male or female animals resulted in similar conclusions.^15^^,^^16^

Despite the discussed limitations, this study addresses key clinical questions regarding early wound healing with SCSA for two early time points. This analysis confirms re-epithelialization as early as 3 days post-SCSA treatment, preceding the formation of a visibly detectable epithelial layer.

## CONCLUSION

Treatment of acute full-thickness porcine wounds with SCSA induced rapid re-epithelialization, with complete closure at the cellular level observed as early as 3 days post-surgery. Histological analyses of biopsied wounds at early time points revealed a dual mechanism of action, with keratinocytes originating both from the wound edges of the interstices and from within the center of the wound bed, contributing to the formation of the initial epidermal layer. Importantly, histological evidence of early re-epithelialization preceded the appearance of a visibly detectable epithelial surface. Through this mechanism, SCSA accelerates wound healing and offers a promising strategy to enhance current acute wound treatment algorithms.

## Notes

Author contributions: Katie A. Bush (Conceptualization [lead], Data curation [equal], Formal analysis [equal], Funding acquisition [lead], Methodology [lead], Supervision [equal], Validation [lead], Writing—original draft [supporting], Writing—review & editing [lead]), Elisa N. Stephens (Visualization [lead], Writing—original draft [lead]), Ghaidaa Kashgari (Data curation [supporting], Formal analysis [equal], Investigation [supporting], Validation [equal], Visualization [supporting], Writing—original draft [supporting], Writing—review & editing [supporting]), Aleisha K. Chamberlain (Visualization [supporting], Writing—original draft [supporting], Writing—review & editing [supporting]), and Steven A. Kahn (Conceptualization [equal], Data curation [equal], Supervision [equal], Writing—review & editing [equal])

Funding: The study was funded by AVITA Medical.
